# Adjuvant radiation therapy for malignant Abrikossoff’s tumor: a case report about a femoral triangle localisation

**DOI:** 10.1186/s13014-018-1064-4

**Published:** 2018-06-20

**Authors:** C. Marchand Crety, C. Garbar, G. Madelis, F. Guillemin, P. Soibinet Oudot, J. C. Eymard, S. Servagi Vernat

**Affiliations:** 1 Department of Radiation Therapy, Institut de Cancérologie Jean Godinot, Reims, France; 2 Department of Pathology, Institut de Cancérologie Jean Godinot, Reims, France; 3 Department of Medical Physic, Institut de Cancérologie Jean Godinot, Reims, France; 4 Department of Surgery, Institut de Cancérologie Jean Godinot, Reims, France; 5 Department of Medical Oncology, Institut de Cancérologie Jean Godinot, Reims, France

**Keywords:** Abrikossoff’s tumor, Granular cell tumor, Treatment

## Abstract

**Background:**

Granular cell or Abrikossoff’s tumors are usually benign however rare malignant forms concern 1 to 3% of cases reported. Pelvic locations are exceptional.

**Case presentation:**

We report a case of a 43-years-old patient who had a benign Abrikossoff’s tumor localized in the right femoral triangle diagnosed at the biopsy. The patient underwent a surgical tumorectomy and inguinal lymph nodes resection. Histologically, the tumor showed enough criteria to give diagnosis of malignancy: nuclear pleomorphism, tumor cell spindling, vesicular nuclei with large nucleoli. Moreover, five lymph nodes were metastatic. Immunohistochemistry findings confirmed the diagnosis of granular cell tumor which is positive for S100 protein and CD68 antibodies. The mitotic index was nevertheless low with a Ki67 labeling index of 1–2%. A large surgical revision with an inguinal curage following radiotherapy were decided on oncology committee. Adjuvant radiotherapy on the tumor bed and right inguinal area of ​​50 Gy in conventional fractionation was delivered with the aim of reducing local recurrence risk. There was no recurrence on longer follow-up (10 months post radiotherapy).

**Conclusions:**

Adjuvant radiotherapy seems an appropriate therapeutic approach, even if controversial, given that some authors report effectiveness on local disease progression.

## Background

Granular cell tumors (GCT), a rare entity, were first described in 1926 by Abrikossoff, at tongue location [1–5]. It is now known that their origin is the schwannian cells [6]. Most of them are benign [7]. GCT are most common in the fourth to sixth decade, but can appear at any age, including children and even congenital cases have been reported [8]. GCT are more frequent in women [4]. Since large series, long-term follow-up studies and oncology protocols and clinical trials on treatment of GCT are lacking, it is not possible to draw firm conclusions on optimal treatment and follow-up procedures of GCT. Local surgical excision with clear margins and Mohs micrographic surgery have been utilized for treatment of benign and malignant GCT [9–11]. The approach for a malignant lesion may also include regional lymph node dissection. The role of radiotherapy and chemotherapy is still uncertain, most reports describing poor response to these two therapeutic modalities [12].

We present, here an unusual case of a patient who was treated for an atypical GCT localized in the right inguinal region with surgery and radiotherapy due to the malignant lesion and the lymph node metastasis.

## Case presentation

**Fig. 1:**
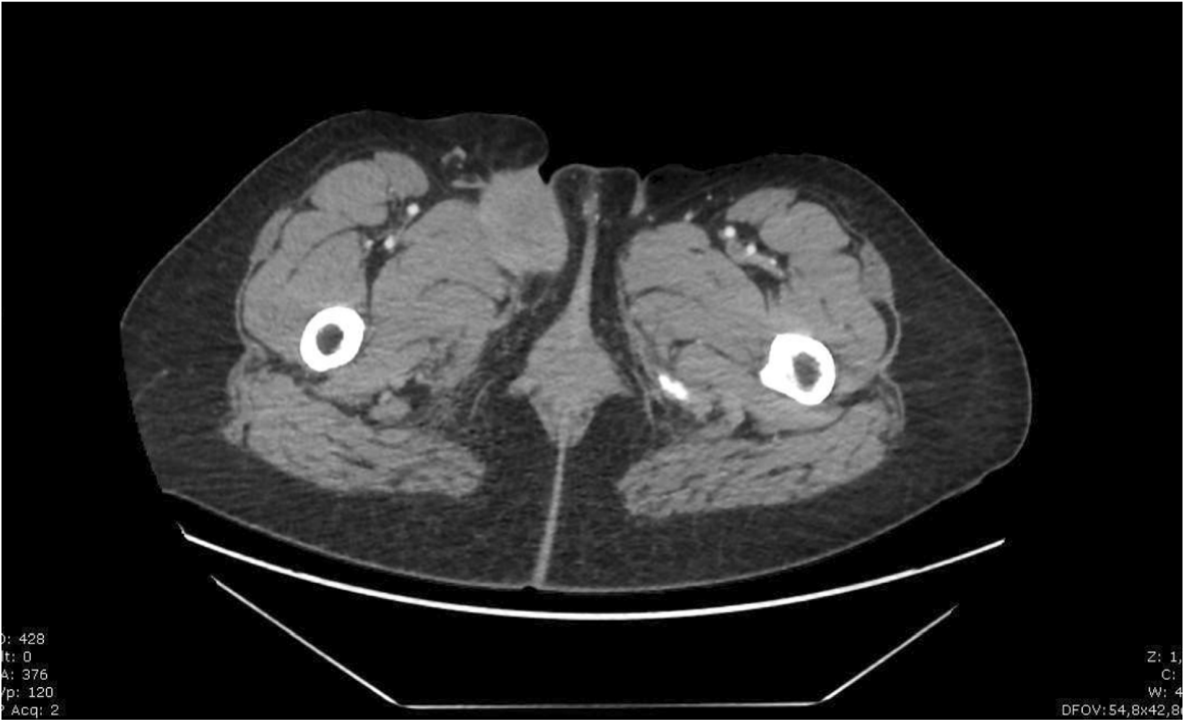
Initial CT scan. Axial CT scan showing an inguinal non-circumscribed contour 5 cm long axis, mass adhering to skin and in contact with the adductor muscle of the deep lip

The patient is a 43-year-old woman who was admitted for the first time for a progressive non-painful, mobile mass of the right inguinal fold evolving for 7 months. The medical history of the patient included childhood asthma, chronic tonsillitis, seven pregnancies and four children, caesarean section and abortions. Pelvic ultrasound showed a heterogeneous suspicious non-circumscribed mass measuring 5 cm in its longer axis. It was localised in the right inguinal region and showed cutaneous adhesions. CT scan confirmed the presence of this inguinal mass, measuring 5.8 × 4.9 × 3.2 cm and extending within the right femoral triangle in contact with the long adductor muscle, without enhanced contrast, and without locoregional lymph node (Fig. 1).

**Fig. 2:**
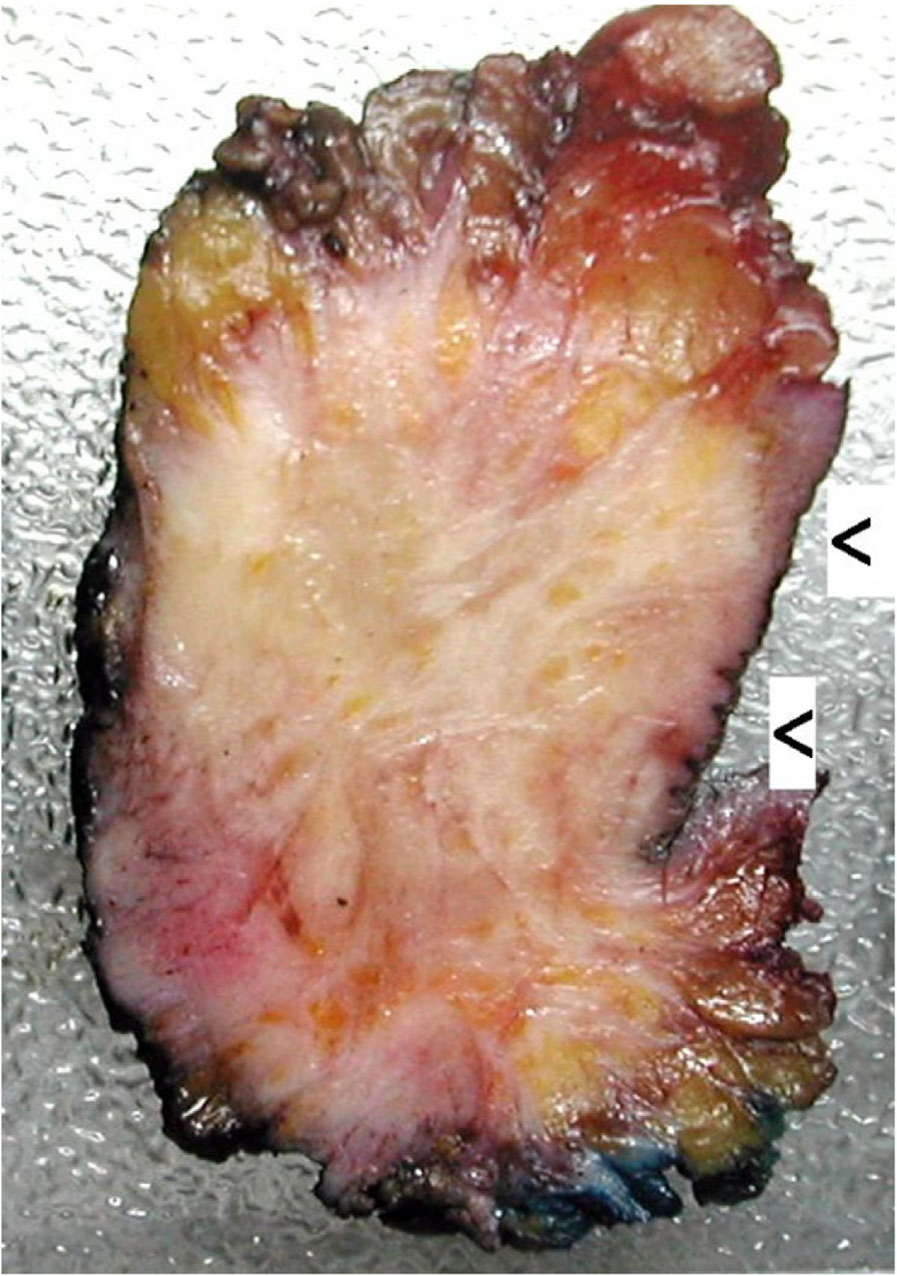
Gross pathology. Poorly defined greyish tumoral mass infiltrating hypodermic and dermic tissues. The epidermis is observed (Arrows)

**Fig. 3:**
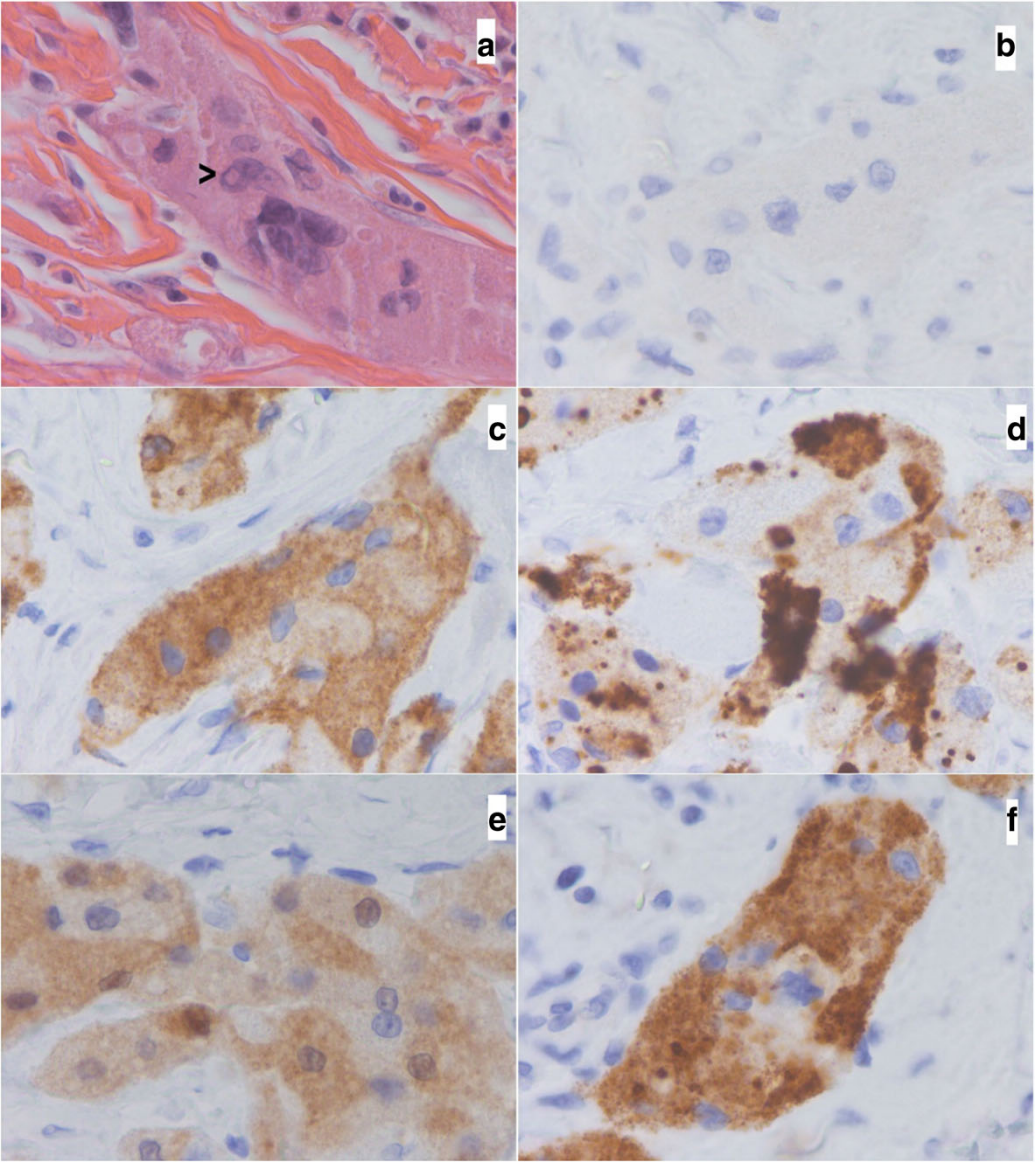
Microscopy and Immunohistochemistry. Microscopic and immunohistochemical findings: **a** Hematoxilin and eosing stain. See nuclear pleomorphim and nuclear vacuole (arrow). **b** Negative expression of cytokeratine AE1-AE3. **c**–**f** Positive expression of CD56, CD57, calretinin and α-inhibin antibodies (magnification 60×)

The patient underwent a chirurgical biopsy. The pathological analysis diagnosed a granular cell tumor (Abrikossoff’s tumor) without any malignant signs (absence of mitosis, necrosis and cytonuclear atypias). Tumorectomy of this inguinal mass were performed three weeks later. At the gross pathology examination, the tumoral tissue was homogeneous with a greyish stain. Its margin was not well defined and the hypodermic, dermic were involved. One lymph node was discovered and was invaded. The epidermis was not ulcerated (Fig. 2). Histologically, collagen bundles were infiltrated by cords of large, polygonal cells with inconspicuous cell membrane and homogenous finely granular cytoplasm. Nuclei were round or oval and presented large nucleoli, vesicular of dark chromatin and sometime an intranuclear vacuole. Mitosis were rare and the mitotic index was low (1 mitosis/ 10 High Power Field). There was a slight increase of the nucleo-cytoplasmic ratio. We observed no necrosis (Fig. 3). Fanburg-Smith score of malignancy was of 3: nuclear pleomorphism, tumor cell spindling, vesicular nuclei with large nucleoli. Immunohistological finding showed a cell expression of S-100 protein, vimentin, calretinin (slight), α-Inhibin, CD56, CD57, CD68 and neuron specific enolase (NSE). (Fig. 3) Cytokeratin AE1-AE3, EMA, calponin, caldesmon, desmin, smooth muscle actine, myosin, myogenin, chromogranin, synaptphysin, Neurofilament proteins, Glial fibrillary acidic protein, CD1a, renal cell carcinoma antibodies were all negative. Therapeutic marker, estrogen receptors, progesterone receptors, androgen receptor, HER2, CD117 ALK, C-MET, ROS1 and PDL-1 were negative too.

Due to microscopic resection and the nodal status, a large surgical revision with an inguinal curage was then decided at the oncology committee. Pathological evaluation did not reveal tumor tissue in the tumorectomy region but showed metastatic invasion by granular cells in four of twelve lymph nodes, without capsular rupture. Adjuvant chemotherapy was excluded because of very low mitotic activity. To reduce the risk of local recurrence, adjuvant radiotherapy on the tumor bed and right inguinal area of ​​50 Gy in conventional fractionation was delivered. After 10 months after the end of radiotherapy, the patient is in complete remission. Due to the unpredictable tumor, the follow up strategy is a physical examination with a CT scan every 4 months for the first 2 post-operative years, then every 6 months for up 5 years and yearly thereafter, as a sarcoma.

## Discussion

Granular cell tumors are rare (0.019 to 0.03% of all cancers) [5]. GCT are most common in the fourth to sixth decade, but can appear at any age, including in children and even congenital cases have been reported [8]. The male/female proportion varies but GCT are more frequent in women [1, 2, 13]. Granular cell tumors mainly develop at the mucocutaneous region. GCT have been reported in many different locations, most commonly in the head and neck region [14], skin [15], subcutaneous and soft tissue [16, 17] and also in the breast [9], thyroid [18], mediastinum [19], respiratory tract [20], gastrointestinal tract [11, 21], female and male genitalia [22, 23], urinary system [24] and peripheral/central nervous system [25]. Among the few pelvic locations, there are some cases in the vulva [23]. No case of inguinal location or within the femoral triangle has been described in the literature.

Clinically, granular cell tumor is generally a protruding nodule, measuring between 1 and 2 cm, painless and firm on palpation. Overlying skin and mucosa may be normal, greyish, yellowish or ulcerated in case of trauma [26]. Tumors with benign behavior, with or without atypical morphology, represent the majority of cases, while malignant GCT with documented metastatic disease comprise less than 3% of GCT. These tumors grow more rapidly and have the potential to metastasize. The most common sites for metastatic spread are the regional lymph nodes, lung, liver and bones.

Currently, neurogenic origin of granular cell tumors is the most likely hypothesis [27]. Existence of tumor connections with nerve branches, as well as the S100 neuroectodermal protein, NSE and myelin proteins expressions, support this theory [8, 26, 28].

Rare malignant forms represent 1 to 3% of cases, clinically suspected by a size greater than 4 cm, necrotic or hemorrhagic plaques or rapid growth [29]. Histologically, Funburg-Smith et al. have developed a more precise classification with six criteria (tumor necrosis, fusiform cells, vesicular nucleus with large nucleoli, mitotic index greater than 2 for ten fields, high nucleocytoplasmic ratio and pleomorphism) [26, 23]. At least three of these criteria are enough to consider the granular tumor as malignant. In our case, invasion of the locoregional lymph nodes allowed to assert the malignancy.

Other authors suggest that rapid growth, tumor size greater than 4 cm, mitoses and high Ki67 (greater than 20%) are in favor of malignancy [13]. Malignant forms require an extension assessment to search for lymphatic or systemic secondary localizations (lung, liver, bone). Indeed, pulmonary secondary injuries are frequently described, and locoregional lymph nodes involvement goes with most malignant tissue lesions. In our case, extension assessment by chest-abdomen-pelvic computerized tomography showed no secondary injury.

Any suspicious soft tissue lesion should be examined by MRI before surgical resection. Standard treatment for granular cell tumors is surgical, requiring complete resection with healthy margins; incomplete resection requires surgical revision (high risk of tumor recurrence) [30]. In malignant forms, safety margins of 2 to 3 cm are certainly recommended because of the risks of local recurrence (32 to 41% of the cases) and metastatic (50 to 63% of the cases) [23, 31]. Benefit of adjuvant radiotherapy, proposed by some authors, is very controversial [12]. However, some observations report radiotherapy efficiency in case of local recurrence or inoperable metastases [27]. In the previous case reports, radiotherapy was use in adjuvant intention after surgery in 6 cases, [12, 27, 32–35] and after local recurrence in 3 cases [36–38]. After adjuvant radiation 2 patients of 6 had developed a local recurrence in field and 1 of them with distant metastasis.

Due to regional lymph node metastases, our patient received adjuvant radiotherapy with dose of 50 Gy to treat the tumor bed and the lymph node region. Moreover, benefit of chemotherapy is not proven, several cases testify to the low chemosensitivity of granular cell tumor. In this case, radiotherapy combined with surgical excision has produced local control, good cosmetic and preserved function. Adjuvant radiotherapy may be appropriate for patients with GCT thought clinically and pathologically to be at high risk of local recurrence or metastasis.

## Conclusion

In conclusion, Abrikossoff’s tumor, although benign in a large majority of cases, must alert as to its potential malignancy. With preferential localizations, this tumor can grow anywhere. Since chemotherapy has not been effective, local adjuvant radiotherapy after a large excision, seem to be an appropriate therapeutic approach in malignant granular cell tumors.

## Abbreviations

Gy: Gray
NSE: neuron specific enolase
